# Evidencing the role of carbonic anhydrase in the formation of carbonate minerals by bacterial strains isolated from extreme environments in Qatar

**DOI:** 10.1016/j.heliyon.2022.e11151

**Published:** 2022-10-20

**Authors:** Rim Abdelsamad, Zulfa Al Disi, Mohammed Abu-Dieyeh, Mohammad A. Al-Ghouti, Nabil Zouari

**Affiliations:** Environmental Science Program, Department of Biological and Environmental Sciences, College of Arts and Sciences, Qatar University, PO. Box 2713, Doha, Qatar

**Keywords:** *Carbonic anhydrase*, Carbonate formation, Extreme environments, Sabkha, Marine sediments, *Virgibacillus*

## Abstract

Calcium carbonate, one of the most abundant minerals in the geological records is considered as primary source of the carbon reservoir. The role of microorganisms in the biotic precipitation of calcium carbonate has been extensively investigated, especially at extreme life conditions. In Qatar, Sabkhas which are microbial ecosystems housing biomineralizing bacteria, have been carefully studied as unique sites of microbial dolomite formation. Dolomite (CaMg(CO_3_)_2_ is an important carbonate mineral forming oil reservoir rocks; however, dolomite is rarely formed in modern environments. The enzyme carbonic anhydrase is present in many living organisms, performs interconversion between CO_2_ and the bicarbonate ion. Thus, carbonic anhydrase is expected to accelerate both carbonate rock dissolution and CO_2_ uptake at the same time, serving as carbonite source to carbonites-forming bacteria. This study gathered cross-linked data on the potential role of the carbonic anhydrase excreted by mineral-forming bacteria, isolated from two different extreme environments in Qatar. Dohat Faishakh Sabkha, is a hypersaline coastal Sabkha, from where various strains of the bacterium *Virgibacillus* were isolated. *Virgibacillus* can -not only-mediate carbonate mineral formation, but also contributes to magnesium incorporation into the carbonate minerals, leading to the formation of high magnesium calcite. The latter is considered as precursor for dolomite formation. In addition, bacterial strains isolated from marine sediments, surrounding coral reef in Qatar sea, would provide additional knowledge on the role of carbonic anhydrase in mineral formation. Here, the quantification of the two mostly described activities of carbonic anhydrase; esterase and hydration reactions were performed. Mineral-forming strains were shown to exhibit high activities as opposed to the non-forming minerals, which confirms the relation between the presence of active carbonic anhydrase combined with elevated metabolic activity and the biomineralizing potential of the bacterial strains. The highest specific intracellular carbonic anhydrase activity; as both esterase and hydration (i.e., 66 ± 3 and 583000 ± 39000 WAU/10^8^ cells respectively), was evidenced in mineral-forming strains as opposed to non-mineral forming strains (i.e., 6 ±. 0.5 and 1223 ± 61 WAU/10^8^cells) respectively. These findings would contribute to the understanding of the mechanism of microbially mediated carbonate precipitation. This role may be both in capturing CO_2_ as source of carbonate, and partial solubilization of the formed minerals allowing incorporation of Mg instead of calcium, before catalyzing again the formation of more deposition of carbonates.

## Highlights

• *Carbonic anhydrase* is involved in mineral formation by *Virgibacillus, Vibrio alginolyticus and Pyschrobacter* strains.
• Both intracellular esterase and hydration carbonic anhydrase activities are exhibited by the mineral forming strains.
• Strong potential exists with bacteria exhibiting high carbonic anhydrase activities to form mineral carbonates.

## Graphical abstract

**Figure:**
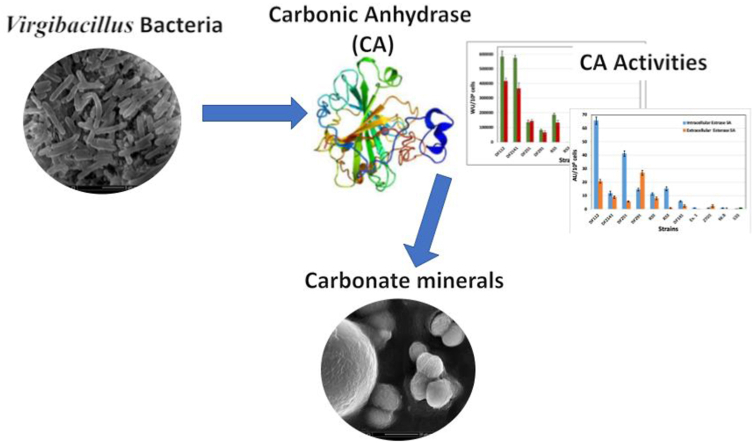

## 1 Introduction

Calcium carbonate, one of the minerals that is largely available among all those formed from geological processes, is a primary source of the carbon reservoir (Romanov et al., 2015). Bacteria and plenty of other organisms are said to be responsible of the biotic precipitation of calcium carbonate, and the process has been extensively investigated (Dhami et al., 2013; Zhu and Dittrich, 2016). Studies have been performed on microbially induced calcite precipitation (MICP) in which the enzyme named urease is involved if urea is available in the surrounding environment (Bibi et al., 2018) or microbially mediated carbonate precipitation (MMCP), in which the exopolymeric substances synthesized by the microbial cells are involved (Al Disi et al., 2017, Al Disi et al., 2019a). Although the mechanism of MICP is almost known, many evidences have led to confirm that the availability of bacterial cells and certain enzymatic activities in a supersaturated medium promotes mineralization of calcium carbonate by MMCP (Bose and Satyanarayana, 2017). Not only do they add to the global primary production and fixation of CO_2_, microorganisms also aid in the processes of lithification and precipitation after interacting with the sediments (Prieto-Barajas et al., 2018).

Nowadays, Sabkhas are vulnerable areas regarding future climate projections, with little rainfall, restricted freshwater supplies and rising population (Cusack et al., 2018). Despite the harsh conditions, mangroves, Sabkhas and seagrasses along the Arabian Gulf seem to serve as CO_2_ sinks (Macreadie et al., 2019), influencing the so-called “blue carbon,” or the carbon stored in marine systems (Duarte et al., 2013). Until now, carbon storage in blue carbon habitats has been excessively studied in temperate and tropical climates (Cusack et al., 2018). The data for arid regions, including the adjoining Arabian Gulf, are very scarce, and the understanding of organic carbon storage in coastal ecosystems remains restricted ((Schile-Beers et al., 2017; Wu et al., 2019). Naturally, microorganisms enhance their growth and sustain their survival by assembling in different forms of aggregates that can be of one species such as in biofilms, or complex well-established ecosystems in the case of microbial mats (Dupraz et al., 2009). In Qatar, Sabkhas which are microbial ecosystems housing biomineralizing bacteria, have been carefully studied. The most significantly mineralizing bacteria isolated from the hypersaline Dohat Faishakh Sabkha, which is a coastal Sabkha located in the northwest of Qatar, belong to the genus *Virgibacillus* along with other aerobic, halophilic and heterotrophic bacteria (Al Disi et al., 2017). Many isolated bacterial strains from Qatar Sabkhas have been identified as *Virgibacillus*; although also reported by Vreeland et al. (2000) and Satterfield et al. (2005) in the Permian Salado salts in the Guadalupe Mountains of western Texas, U.S. Thus, the Sabkhas of Qatar are today considered as ubiquitous environment for the investigation of the role of aerobic bacteria, specifically *Virgibacillus*, in mineral precipitation (Al Disi et al., 2017). It is now well established, that *Virgibacillus* is not only responsible of mineral formation, but also contributes in the incorporation of magnesium into the carbonate crystals, leading to the formation of high magnesium carbonates and protodolomite that are considered as precursors for dolomite formation (Gregg et al., 2015). This function is unique among microorganisms. However, the mechanism of incorporation of magnesium is still not well elucidated, as well as the source of carbonate employed by the bacterial cell to form the high Mg-calcite minerals.

In such an interest, it has been frequently reported in literature that both enzymes; urease and carbonic anhydrase are critical for proficient mineralization of calcium carbonate and other minerals (Achal and Pan, 2011). Hydrolysis of urea, available in the microenvironment of the bacterial cells, by urease leads to carbonate production (Bibi et al., 2018). *Carbonic anhydrase*, being far less studied, seems a very interesting potential area of investigation to many scientists (Dhami et al., 2014). The carbonic anhydrase enzyme itself is present in many living organisms (eukaryotes, prokaryotes, archaea) and it works on catalyzing the reversible hydration of carbon dioxide (Lionetto et al., 2016). Actually, a debate is still ongoing regarding the mineralization process as a side effect of microbial metabolism under certain conditions or a direct effect based on the response of the microorganisms to environmental factors. The essential abiotic parameters that regulate precipitation of minerals are known to be: (1) concentration of Ca^2+^, (2) pH of the medium/environment, (3) dissolved inorganic carbon (DIC), (4) nucleation sites availability. However, it is still questionable whether the bacterial carbonic anhydrase plays a role in the mineralization process. The enzyme is known to be a zinc-containing metalloenzyme, catalyzing the reversible reaction between CO_2_ and HCO_3_^−^ (CO_2_ + H_2_O ⇔ HCO_3_^-^ + H^+^) (Bose and Satyanarayana, 2017; Occhipinti and Boron, 2019). Thus, this enzyme is able to accelerate both carbonate rock dissolution and CO_2_ uptake at the same time. Previously, tests with rotating-disk or shaking flask as well as with soil columns were performed for the simulation and the investigation of several biological factors, including carbonic anhydrase activity involved in dissolution of carbonate rock (Supuran, 2013). However, the CO_2_ capture was not clearly elucidated. Moreover, Xiao and Lian (2016) specifically studied the expression of carbonic anhydrase from *Bacillus* sp. in *E. coli*. Their findings showed that the microbial carbonic anhydrase aided in capturing the atmospheric CO_2_. However, it is important to investigate to what extent microbial carbonic anhydrase is involved in the capability of *Virgibacillus* strains isolated from the Qatar environment in minerals formation. It is anticipated that the carbonic anhydrase reaction will provide the fundamental molecules required for the formation of CaCO_3_ when taking place in the forward direction. Nevertheless, the role of this enzyme in CaCO_3_ precipitation is still ambiguous. Indeed, the marine actinobacterium strain *Brevibacterium linens* BS258 was responsible of forming precipitates of calcium carbonate using the urease activity (Zhu et al., 2017). The same authors also showed that this strain dissolves the formed precipitates when the calcium concentration was increased. They finally attributed this role to carbonic anhydrase activity as it was overexpressed at high calcium concentration (Zhu et al., 2017).

In an attempt to draw a correlation between calcium carbonate precipitation and carbonic anhydrase activity in biomineralizing bacterial strains, the present work intends to investigate the activities of carbonic anhydrase in mineral forming *Virgibacillus* spp. strains and non-mineral forming strains, all isolated from Dohat Faishakh Sabkha in Qatar. As a first inquiry, it is of interest to quantify the carbonic anhydrase activity to link it to a possible role of the cell surface in mediating biomineralization. Studying the influence of carbonic anhydrase and elevated metabolic activity on *Virgibacillus* capabilities to mediate carbonate minerals formation would provide a clear answer of the potential source of carbonate for the cell to form calcite. Recently, 35 mineral forming bacterial isolates isolated from Dohat Faishakh Sabkha were categorized by MALDI-TOF MS combined to PCA (Abdel Samad et al., 2020). Specific proteins biomarkers were identified among the mineral forming bacterial strains when cultured in appropriate media. In order to gather cross-linked data on the potential role of the carbonic anhydrase in bacteria, another extreme environments were considered. Indeed, bacteria isolated from marine sediments, surrounding coral reef in Qatar sea, would provide additional knowledge if mineral-forming bacteria can be involved in marine mineral formation and if carbonic anhydrase is also associated. Indeed, marine sediments were sampled from sites close to the coral reef in Qatar, to isolate such type of bacteria.

The carbonic anhydrase activity is reported with two measurable activities, esterase and hydration activities (Angeli et al., 2020). Both activities should be checked to evidence if they are involved in mineral-formation by mineral-forming strains as opposed to non-mineral forming strains. These findings would contribute to the elucidation of the mechanism of microbially mediated carbonate precipitation. This role may be both in capturing CO_2_ as source of carbonate, and partial solubilization of the formed minerals (Zhu et al., 2017; Oualha et al., 2020). Indeed, it is now claimed that one of the most possible mechanisms allowing incorporation of Mg^2+^ instead of Ca^2+^ in dolomite formation, is the partial solubilization of carbonates before catalyzing again the formation of more deposition of carbonates with substitution of Ca^2+^ by Mg^2+^ (Hao et al., 2020).

## 2 Material and methods

### 2.1 Bacterial strains (reference strains)

Four *Virgibacillus* strains and one *Bacillus licheniformis* strain were used as reference strains in this work. These strains were previously isolated from decayed mats of Dohat Faishakh Sabkha in Qatar and identified by ribotyping by Al Disi et al. (2017). The other strains were isolated from soils in Qatar ((Bibi et al., 2018; AlKaabi et al., 2020). Table 1 shows the used bacterial strains, their MALDI-TOF MS scores and their identification by ribotyping (the sequences were deposited in the NCBI database, and their access number are provided). The *Virgibacillus* strains were not identified by MALDI-TOF MS, because the database was missing protein profiles of this genus.

**Table 1:** Bacterial strains from decaying mat sampled from Dohat Faishakh Sabkha and soils in Qatar.

Strain code	Origin	Identification by ribotyping	NCBI Accession Number	MALDI-TOF score	Mineral forming on MD1	Reference
DF112	DF Sabkha	*Virgibacillus marismortui*	KY361738	NA	+	(Al Disi et al., 2017)
DF2141	DF Sabkha	*Virgibacillus* sp.	KY360309	NA	+	(Al Disi et al., 2017)
DF251	DF Sabkha	*Virgibacillus* sp.	KY365009	NA	+	(Al Disi et al., 2017)
DF291	DF Sabkha	*Virgibacillus* sp.	KY359388	NA	+	(Al Disi et al., 2017)
DF141	DF Sabkha	*Bacillus licheniformis*	KY363571	1.81	-	(Al Disi et al., 2017)
S33	Soil	*Bacillus licheniformis*	-	2.07	-	-
Z7D1	Soil	*Virgibacillus halodenitrificans*	KT945027.1	NA	-	(AlKaabi et al., 2020)
6.8	Soil	*Bacillus cereus*	GQ495095.1	2.12	-	(Bibi et al., 2018)

### 2.2 Samples of marine sediments

**Figure 1:**
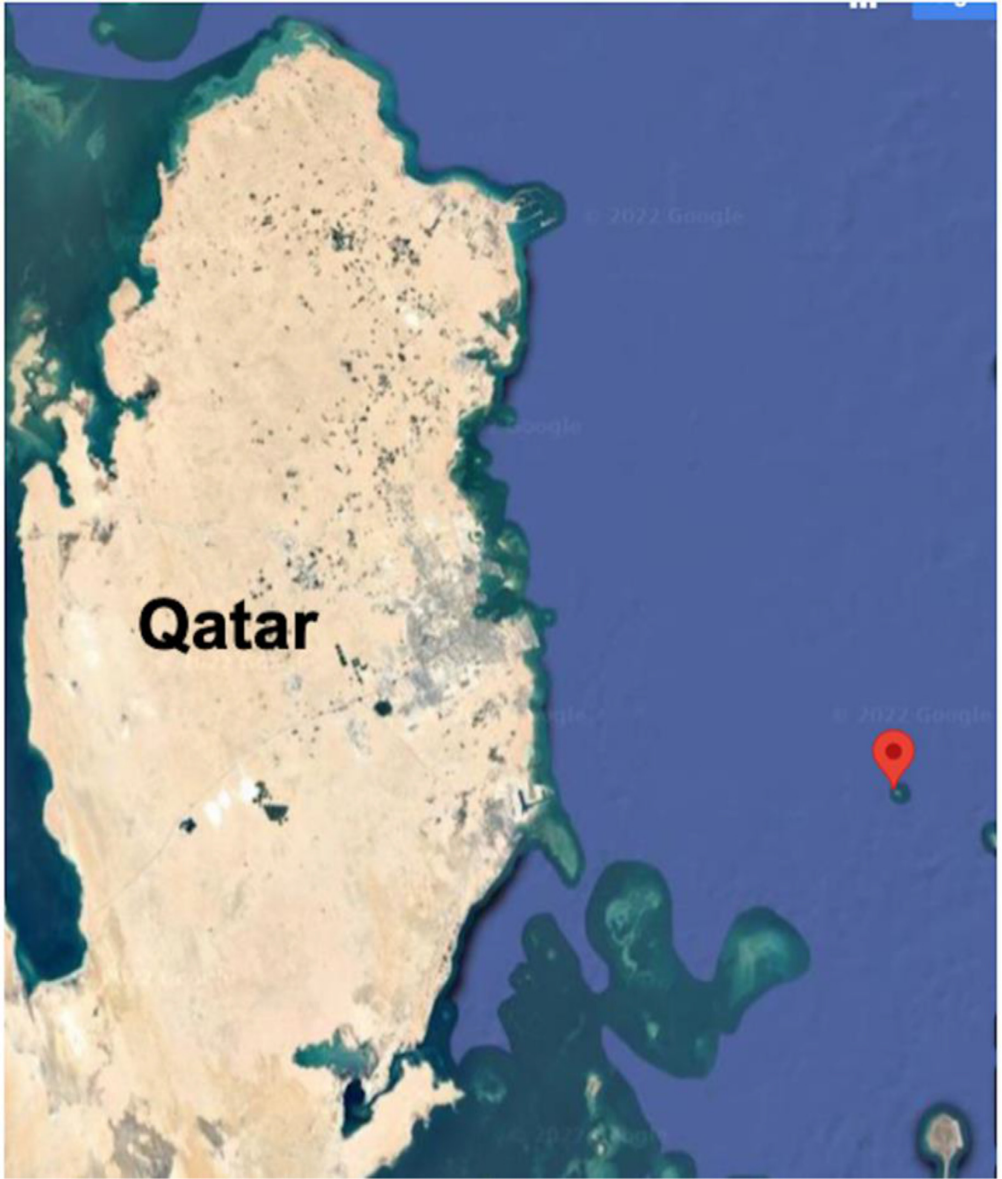
Map of Qatar showing sampling point.

50 g of sediment samples were harvested in sterile 50 ml Falcon tube, from Qatar marine environment in December 2019. The sampling was performed in the Arabian Gulf (GPS coordinates 25°03213"N, 52°22474"E) (Figure 1). The samples collected from the surface at depth of 5 cm were temporally stored in an icebox at 4 °C and then transferred to the laboratory for further analysis. They were stored at -20 °C for three days before they were used for enrichment culturing. The enrichment cultures were performed by suspending 1 g of sediments in 20 ml liquid MD1 medium. The cultures were then incubated for 48 h in the shaker set at 30 °C and 150 rpm. Consequently, four successive enrichment cultures were performed at the same conditions, each inoculated using 2 ml from the previous culture.

### 2.3 Culture media

MD1, MD1-, MD2 and MD3 media were used in this study. The MD1 medium is composed of (% w/v): 1 yeast extract, 0.5 peptone, 0.1 glucose and 3.5 NaCl. It is supplemented with acetate salts, which are Ca(C₂H₃O₂) ₂ and (CH_3_COO)_2_Mg.4H_2_O to obtain the Mg^2+^: Ca^2+^ molar ratios of 6. MD1-is a modified MD1 medium from which glucose was omitted. The medium MD2 is missing yeast extract, and MD3 missing peptone compared to MD1. All the media were sterilized by autoclaving at 121 °C for 20 min. The pH was adjusted to 7 with 0.1 M KOH before sterilization.

### 2.4 Isolation of the bacterial strains

After four successive enrichment cultures in MD1, starting with 1 g of sediment in 20 ml liquid medium and followed by inoculation of the successive cultures with 2 ml per 20 ml, serial dilutions up to 10^−4^ of the fourth culture were plated on solid MD1 and then incubated overnight at 30 °C. The incubation of the liquid cultures was performed at 30 °C in a shaker set at 200 rpm. The light microscopic observations of the bacterial cells of each colony, as well as the colony form and color were used to select the most representative colonies, as formed at the highest dilution of 10^−4^.

### 2.5 Identification of the isolated bacterial strains from marine sediments

The isolated strains were identified by MALDI-TOF MS as described by Abdel Samad et al., (2020). The generated proteins mass spectra by MALDI-TOF MS were analyzed for similarities to the available proteins profiles in the database entries. A log (score) is generated, by the Biotyper software by default, for each strain and used in the identification. Proteins with a m/z between 2000 and 20 000 m/z are used to generate the protein profiles for each strain. The mass peaks corresponding to specific ribosomal proteins are those used for the identification of the isolates by similarities establishment.

### 2.6 Evaluation of the mineral formation by the isolated bacterial strains

MD1, MD1-, MD2 and MD3 solid media were used to investigate the potential of the isolates to form minerals as described by Al Disi et al., (2017). Each isolate was inoculated on the solid media and then incubated at 30 °C for a period of 3 weeks. The inculcated plates were checked periodically using light microscope to detect the formation of crystals.

### 2.7 Investigation of carbonate minerals formation

The formed minerals were recovered from pure cultures of the mineral-forming strains as described by Al Disi et al., (2017). The recovered minerals were analyzed by SEM/EDS and XRD.

SEM images were obtained using Nova Nano Scanning Electron Microscope equipped with Bruker EDS Detector with five nm resolution and a magnification of 200,000×.

The bulk mineralogical composition of the recovered minerals was determined using a PANalytical-multipurpose Empyrean X-ray diffractometer. The Mg mol% of carbonate minerals were calculated according to the position of the XRD (d104) peak of high magnesium calcite using the formula of Goldsmith et al. (1961).

### 2.8 Determination of carbonic anhydrase enzymatic activity

#### 2.8.1 Sample preparation

Cultures were prepared using the MD1 medium, each inoculated with one isolate. The bacterial cells inoculum was prepared by culturing overnight a colony in 15 ml MD1 medium. Then, the optical density at 600 nm (OD_600_) of the inoculum was determined and served to inoculate a 50 ml MD1 for an initial OD_600_ of 0.15. The cultures were incubated in a shaker set at 200 rpm and 30 °C. Periodically (24 h, 48 h, 72 h, 96 h and 120 h), 1 ml was transferred into a sterile Eppendorf tube, centrifuged for 5 min at 8000 rpm, the supernatant transferred into a new tube, and the pellet washed twice with 300 μl 0.1M phosphate buffer (PBS) pH 7.4. Then the pellet was suspended in 1 ml of the same buffer.

#### 2.8.2 Esterase activity determination

The esterase activity assay was performed, following Ramanan et al. (2009). The enzyme reacts with p-nitrophenyl-acetate (p-NPA) to produce p-nitrophenol and acetate, producing a yellowish color. Hence, the esterase activity of each bacterial strain was investigated with both the cells and the supernatant. 250 μl of the tested sample was added to 750 μl of 0.1M PBS buffer pH 7.4. At the moment of the addition of p-NPA as the substrate, which is the beginning of the reaction, the absorbance is measured over the course of 10 min at 405 nm. A calibration curve with different concentrations of p-NPA in the reaction medium was performed to calculate the production of the yellowish color determined by optical density of the assay. p-NPA is a very unstable substrate, indeed, background controls were performed in parallel, to subtract the spontaneous hydrolysis of p-NPA if any (De Caro, 1986). One esterase activity unit is defined as the amount of enzyme that produces 1 μmol *p*-nitrophenol per minute at room temperature. The p-NPA stock solution for esterase activity determination was freshly prepared by dissolving 54 mg in 5 ml of ethanol with strong agitation and stored at 4 °C for a period not exceeding 1 h, then replaced by a new fresh solution. Later, 1.5 ml of this stock solution was added to 8.5 ml of distilled water for the experimental use. All measurements were carried out in triplicate, and values are reported as the mean of the data.

#### 2.8.3 CO_2_ hydration assay (Wilbur–Anderson assay: WA assay)

The WA assay was carried out by mixing 100 μl of the solution containing the enzyme, 12.0 ml Tris-HCl buffer solution (20 mM, pH 8.3), and 8.0 ml of CO_2_ saturated water (Wilbur and Anderson, 1948; Mustaffa et al., 2017; Hou et al., 2019). The CO_2_ saturated solution was prepared by bubbling CO_2_ into MillQ water placed in an ice bath for 3 h. The timer was set once the CO_2_ water was added to the solution (start of the reaction), and the time taken for the pH to drop by 1 unit (to pH 7.3) was recorded. The control solution was Tris–HCl buffer (pH 8.3) without any enzymatic solution. Three replicates were performed, and the average was used in the calculations. The formula used to quantify CO_2_ hydration activity is:

(T_c_ − T_test_) / T_test_

Where:

T_c_: Time needed for one-unit pH drop in the absence of carbonic anhydrase.

T_test_: Time needed for one unit pH drop in the presence of carbonic anhydrase.

The hydration activity was then quantified as follows: each unit of CO_2_ hydration activity corresponds to the amount of carbonic anhydrase required to decrease the pH of the buffer by 1 unit from 8.3 to 7.3, which is expressed as WA units per unit of volume.

## 3 Results

### 3.1 Isolation and identification of the bacterial strains isolated from marine sediments

In recent years, the matrix-assisted laser desorption ionization, time to flight mass spectrometry (MALDI-TOF MS) has become a potential tool for identifying and diagnosing microbes as well as their differentiation. MALDI-TOF MS is inexpensive, rapid and requires simple sample preparation procedures and laboratory infrastructure (Feucherolles et al., 2019). It allows to compare the strains based on specific ribosomal proteins (for identification based on established databases) and total proteins profile (for categorization, PC analysis and study of diversity within the same genus or even species) (Reeve et al., 2018; Feucherolles et al., 2019; Abdel Samad et al., 2020). Eleven strains were isolated and identified from the marine sediments (Table 2).

**Table 2:** Isolated and identified bacterial strains.

No.	Strain code	MALDI Score	Most probable similar genus and species
1.	RS1	1.74	*Exiguobacterium arantiacum*
2.	RS2	1.90	*Exiguobacterium arantiacum*
3.	RS3	2.09	*Vibrio alginolyticus*
4.	RS4	2.30	*Photobacterium damselae*
5.	RS5	1.73	*Pyschrobacter* sp.
6.	RS6	1.98	*Vibrio alginolyticus*
7.	RS7	2.05	*Vibrio alginolyticus*
8.	RS8	1.86	*Vibrio alginolyticus*
9.	RS9	2.23	*Vibrio alginolyticus*
10.	RS10	2.06	*Exiguobacterium arantiacum*
11.	RS11	1.90	*Exiguobacterium arantiacum*

### 3.2 Investigation of the mineral-forming potentials of the marine bacterial isolates

Using different media known to be appropriate to the mineral-forming bacteria to deposit carbonate minerals, the ability of all the marine bacterial isolates to form minerals was investigated. Since the growth medium MD1 has been previously reported to mediate precipitation by *Virgibacillus* species from decaying Sabkha mats, it was the first medium on which the isolates were sub-cultured and incubated for 20 days at 30 °C, as described by Al Disi et al. (2017). Subsequently, MD1-, MD2 and MD3 media were used for further investigations of the mineral forming potentials. Indeed MD1-, MD2 and MD3 are made by specific alteration to the MD1 by removing either glucose, or peptone or yeast extract components. During our study, we observed that some bacterial strains might mediate mineral formation only when grown on media, modified according to these alterations of MD1 (data not shown). Nine representative marine bacterial isolates four *Exiguobacterium arantiacum* (RS1, RS2, RS11 & RS12*,* four *Vibrio alginolyticus* (RS3, RS7, RS8 & RS10) and one *Psychrobacter* sp. (RS5) were selected. Table 3 illustrates the results of the investigation of the mineral forming bacterial strains isolated from the marine samples.

**Table 3:** Investigation of the potentials of mineral formation by the bacterial strains isolated from the marine samples.

Isolate ID	Strain Name	Mineral forming			
		MD1	MD1-	MD2	MD3
**RS1**	*Exiguobacterium arantiacum*	-	-	-	-
**RS2**	*Exiguobacterium arantiacum*	-	-	-	-
**RS11**	*Exiguobacterium arantiacum*	-	-	-	-
**RS12**	*Exiguobacterium arantiacum*	-	-	-	-
**RS3**	*Vibrio alginolyticus*	**+**	**(++)**	-	-
**RS7**	*Vibrio alginolyticus*	-	NG	NG	NG
**RS8**	*Vibrio alginolyticus*	-	-	NG	NG
**RS10**	*Vibrio alginolyticus*	-	NG	NG	NG
**RS5**	*Psychrobacter* sp.	**+**	**+**	**+**	**+**

(NG) no growth; (-) growth with no precipitation; (+) growth with precipitation; (++) growth with significantly higher precipitation.

### 3.3 Determination of the mineral composition

**Figure 2:**
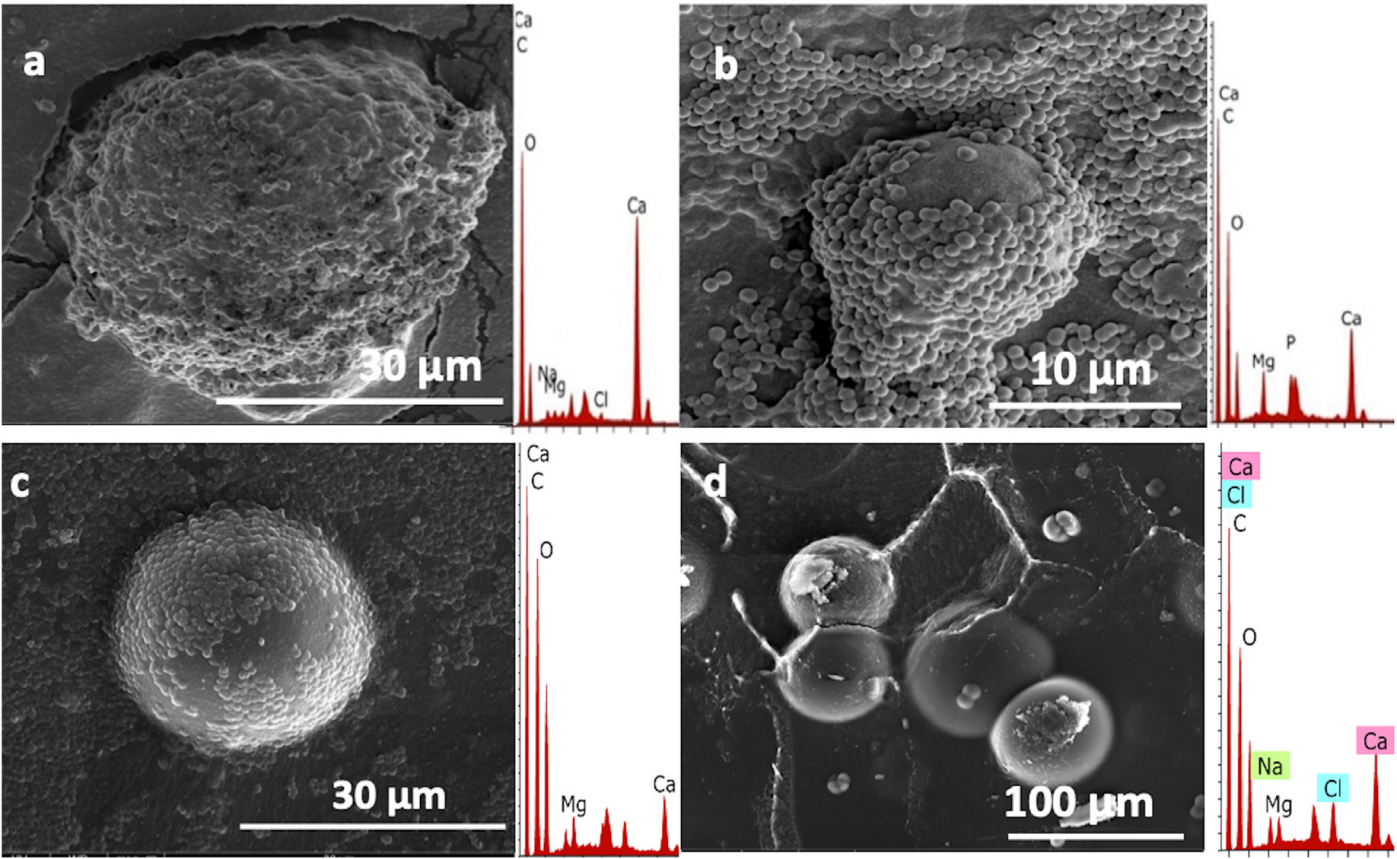
SEM/EDS analysis of crystals formed by A) *Vibrio alginolyticus* (RS3) on MD1, B) and *Vibrio alginolyticus* (RS3) on MD1-. Bacterial cells are shown to be surrounding the crystal with only part of it appearing to be smooth and spherical, C) *Psychrobacter* sp. (RS5) strains on MD3, the crystal formed appears to be a perfect sphere, partially covered with bacterial cells, D) by *Psychrobacter* sp. (RS5) strains on MD2.

**Figure 3:**
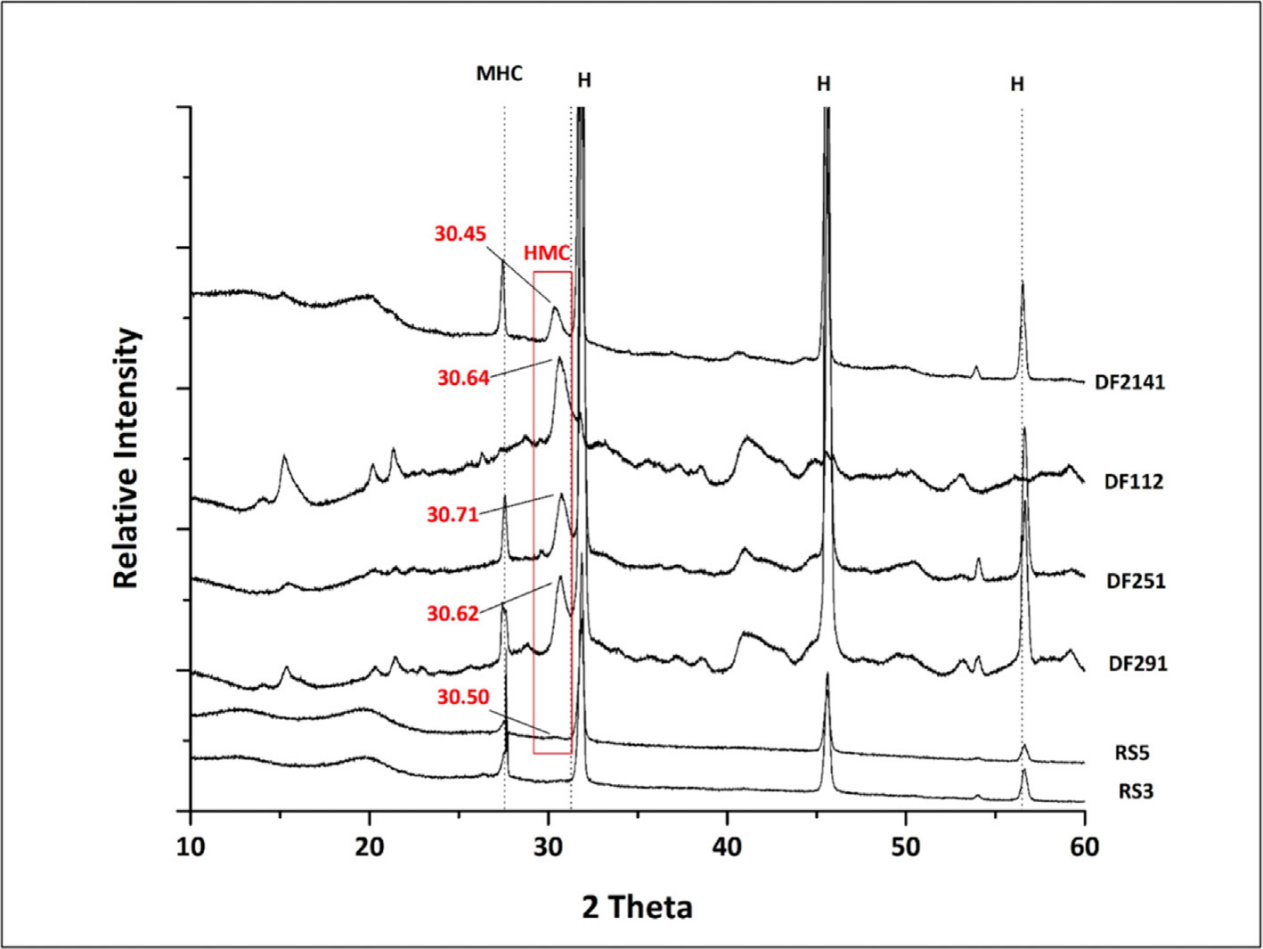
XRD patterns of minerals formed by the strain of *Psychrobacter* sp. (RS5) on MD1 medium compared to minerals formed by *Virgibacillus* reference strains. H: Halite, MHC: Monohydrocalcite, HMC: High Magnesium Calcite.

The association between the growth of each of the mineral-forming bacterial strain and the composition of the formed crystals was determined using the analysis of the minerals by SEM/EDS analysis. Figure 2A shows spherical shaped crystals formed by *Vibrio alginolyticus* (R9) on MD1. The corresponding EDS indicates that they are composed of calcium carbonates with low Mg incorporation. Similar findings have been found with *Vibrio alginolyticus* (RS3), but with higher Mg^2+^ incorporation in the crystals compared to that of the strain *Vibrio alginolyticus* (R9) (Figure 2B). Figure 2C shows spherical crystals formed by the *Psychrobacter* sp. (RS5) strain on MD3 medium, i.e., without peptone, with higher Mg incorporation compared to the crystals formed by *Psychrobacter* sp. (RS5) on MD2 (Figure 2D). Both SEM images shows crystals with smooth surfaces. XRD analysis of the recovered carbonate crystals revealed that the most abundant carbonate precipitates formed by *Psychrobacter* sp. (RS5) were monohydrocalcite. The reference strains used in this study evidenced the very high magnesium calcite phases. Figure 3 illustrates comparisons between the XRD patterns of minerals formed by the studied strains. Table 4 indicates the Mg Mol% in the formed high magnesium calcite phases. Although it is expected that an amorphous phase of the carbonates may have formed, however, no amorphous forms were observed by SEM/EDS or by XRD analyses.

**Table 4:** Carbonate minerals formed by the mineral forming strains and Mg Mol% in the formed HMC minerals.

Strain	Carbonate Minerals formed	Mg Mol%
*Virgibacillus marismortui* (DF112)	High Magnesium Calcite	33.35 ± 1.6
*Virgibacillus* sp. (DF251)	Monohydrocalcite, High Magnesium Calcite	41.52 ± 1.3
*Virgibacillus* sp. (DF291)	Monohydrocalcite, High Magnesium Calcite	38.59 ± 1.4
*Virgibacillus* sp. (DF2141)	Monohydrocalcite, High Magnesium Calcite	35.50 ± 2.1
*Vibrio alginolyticus* (RS3)	Monohydrocalcite	-
*Psychrobacter* sp. *(*RS5)	Monohydrocalcite, High Magnesium Calcite	35.97 ± 1.5

### 3.4 Evidencing the role of carbonic anhydrase and elevated metabolic activity in mineral formation

**Figure 4:**
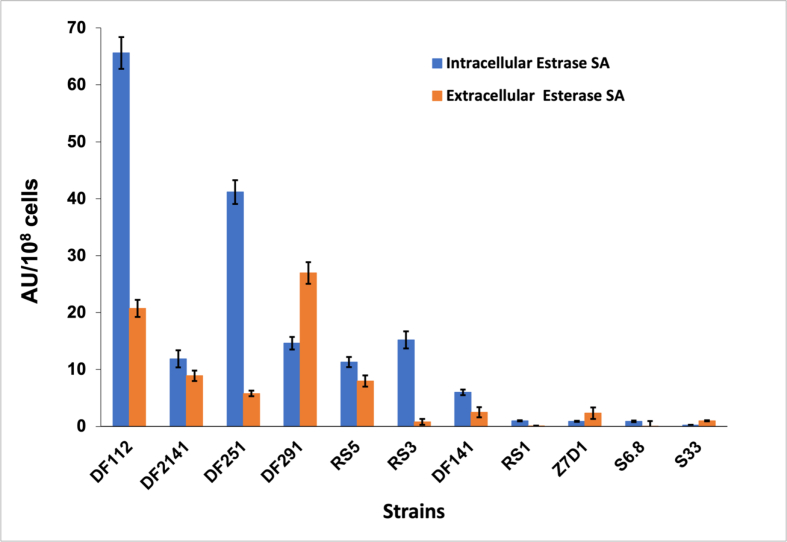
Highest intracellular and extracellular esterase specific activities for the studied strains recorded after 72 h of incubation.

**Figure 5:**
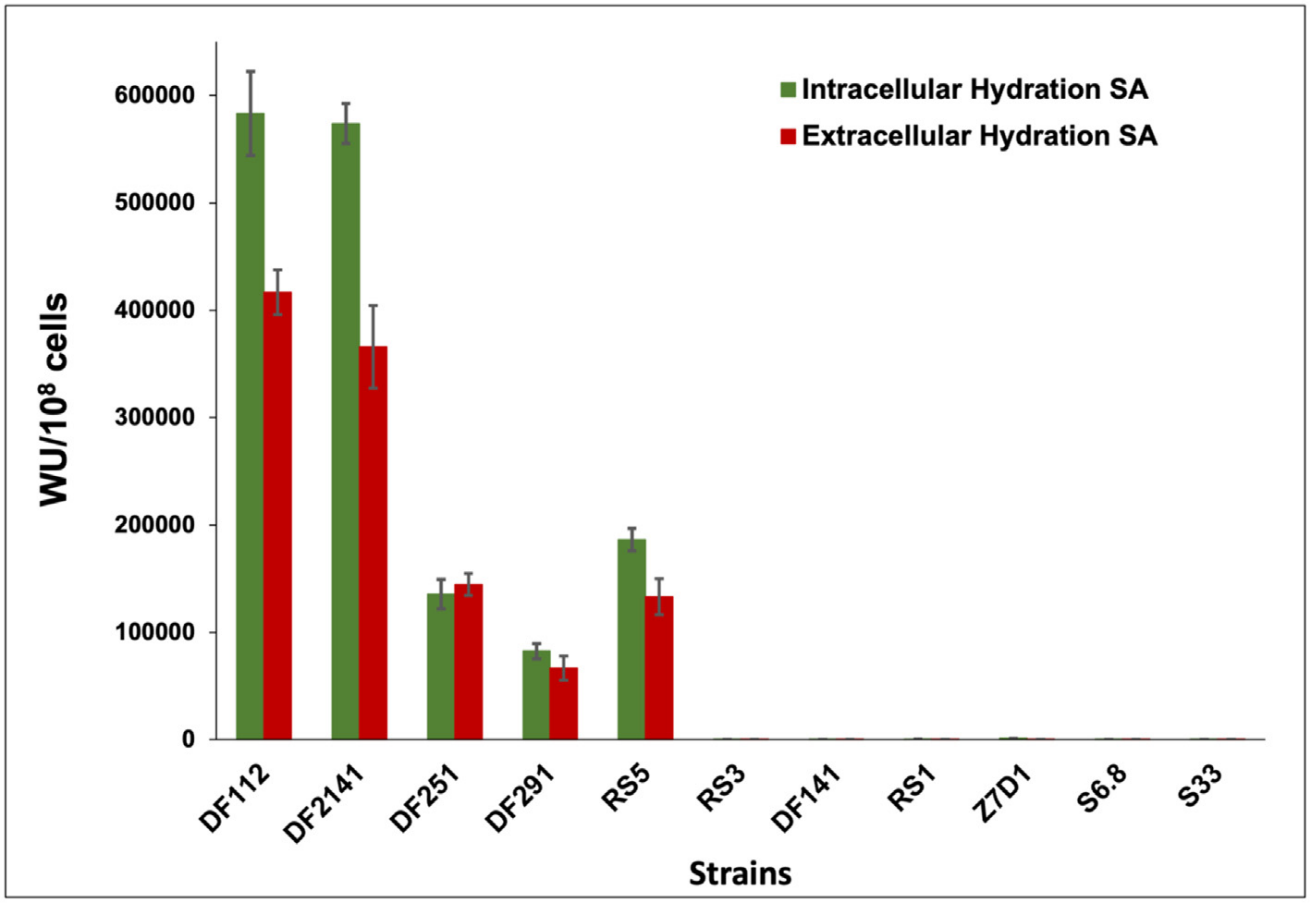
Highest intracellular and extracellular hydration specific activities for the studied strains recorded after 72 h of incubation.

The results of esterase and hydration activities are shown in Figures 4 and 5, respectively. The obtained results are in coherence with those of the mineralization capabilities. The highest intracellular hydration specific activity for the non-mineral forming strains was reported to be 1233 AU/10^8^ cells which is much lower than that obtained with the mineral-forming strains. The intracellular hydration specific activity of the mineral-forming strains ranged from 82609 - 583333 AU/10^8^ cells which is 67–473 folds higher than that of the non-mineral forming ones.

## 4 Discussion

Four of the isolated and identified bacterial strains were *Exiguobacterium arantiacum* (RS1, RS2, RS11 & RS12) and five were *Vibrio alginolyticus* (RS3, RS6, RS7, RS8 and RS9). Although several of both bacteria genera were reported in the literature to be potentially pathogenic to animals, they also play a role in biomineralization (Rivadeneyra et al., 1994). Each strain is characterized by a MALDI log score, in the range of 0–3, as per the manufacturer’s manual. The score of 1.7 to around 1.99 is a relation to the genus level, a score of 2–2.2 is a relation to the genus and species level and a score of 2.3–3 is a relation to the species level. A score lower than 1.7 cannot be used for the identification because it is not so reliable*. Exiguobacterium* is a genus of *Bacillaceae,* associated with low G-C phyla. *Exiguobacterium aurantiacum* is the first strain identified from alkaline potato processing plant (Vishnivetskaya T. A., 2005). They are Gram-positive motile, facultative anaerobes with catalase, not oxidase and not spore-forming. According to Pandey (2017), the *Exiguobacterium* bacterium was also isolated from soils, chemically contaminated wastes, uranium ore, rivers, seawater, carbonate hot spring water, etc. *Exiguobacterium* can survive using an extensive variety of nutrients. The other identified bacterial strains belong to *Vibrio alginolyticus*, a Gram-negative marine bacterium (Kechker, 2017). The bacterium *Vibrio* is known to form Mg^2+^: Ca^2+^ mineral precipitates (Rivadeneyra et al., 1994).

One of the isolated bacterium (RS5) belongs to *Psychrobacter* genus. Vishnivetskaya et al. (2000) isolated the strain *Psychrobacter arcticus* 273–4 from a core, sampled in the extreme environment of Siberian permafrost, which is known to be 20,000 to 40,000 years old (Vishnivetskaya et al., 2000; Bakermans et al., 2006). In addition to the constant temperature of almost −10 °C, the Siberian permafrost is characterized by low nutrients and unfrozen water (Steven et al., 2006). *Psychrobacter* bacterium is also isolated from a variety of terrestrial and marine environments, such as soil, seawater, ice and air (García-López and Maradona, 2000; Batt and Batt, 2014). *Psychrobacter* is considered as ubiquitous bacterium, which can grow at a large temperature range of −10 to 42 °C and resist to ionizing irradiation (Teixeira and Merquior, 2014). A strain of *Psychrobacter* sp. SHUES1 was isolated from soil samples in China and showed very remarkable activity in the precipitation of metals and minerals at low temperatures, and when sequenced, its DNA showed the presence of the gene of the enzyme carbonic anhydrase (Xuezheng et al., 2010; Li et al., 2016).

One of our isolated strains (RS4) belongs to *Photobacterium* damsela, which is a marine *Vibrio*, characterized as halophilic in tropical and semitropical aquatic environments (Rivas et al., 2011). This bacterium was discarded from our collection because it can be associated to severe infection in fish, sharks, and other marine animals (Fouz et al., 2000; Romalde, 2002). In addition, this bacterium is especially known to be infectious and pathogenic to humans, causing severe infections to wounds and necrotizing fasciitis, and can be fatal if not treated at early stage of infection (Rivas et al., 2013).

None of the *Exiguobacterium arantiacum* isolated strains have shown mineralization activity on neither of the media tested, although other species belonging to the genus *Exiguobacterium* also isolated from marine environments have been recently reported to biomineralize and exhibit enzymatic activities that induce mineral formation under high salt stress conditions (Bansal et al., 2016). However, this does not exclude that the isolated strains of *Exiguobacterium arantiacum* would have capability to mineralize at specific biotic conditions. In addition, since they belong to psychrophilic bacteria, they may have enhanced abilities of biomineralization at lower temperatures (Ehrlich and Nikolaev, 2017).

*Vibrio alginolyticus* isolated strains showed a variation of mineralization potential with the different culturing media used. *Vibrio alginolyticus* (RS9) and *Vibrio alginolyticus* (RS3) are interesting because they can form minerals significantly on MD1-medium. Moreover, the only bacterial strain that showed consistent precipitation capability in all the tested 4 media is *Psychrobacter* sp. (RS5). Another *Psychrobacter* sp. SHUES1 isolated from soil samples in China has also showed very remarkable activity in the precipitation of metals at low temperatures, and when sequenced, its DNA showed the presence of a carbonic anhydrase gene (Xuezheng et al., 2010; Li et al., 2016).

In order to investigate the role of the carbonic anhydrase in the biomineralization potential of the mineral-forming bacterial strains, their enzymatic activities were evaluated either by assessing the esterase activity and/or the hydration activity. The methodological approach employed for this study is based on the assumption that the strains used for the experiments produce similar EPS when they are grown in liquid or solid media. In the current research, it was necessary to link such mineral formation conditions to carbonic anhydrase activities. As a consequence, it seems that the difference in incubation conditions has no significant effect on the relationships observed between the mineral formation and the enzyme activities for each strain (Al Disi et al., 2019b). It is to be noticed that the protocol employed in this research determined the elevated metabolic activity involving a combination of esterase activity and proton production such as that achieved by the enzyme carbonic anhydrase in the sample.

Exhibiting the esterase activity, the carbonic anhydrase enzyme acts on carbonyl-compounds, such as ester, by catalyzing the hydrolysis of the ester in a mechanism that is not quite known yet. However, there is enough evidence to conclude that the esterase activity is exhibited by the carbonic enzyme (Elleby et al., 1999). Here, the study was performed using the p-NPA protocol. By the hydration activity, the enzyme is able to release H^+^ upon the production of bicarbonate. Consequently, the enzyme catalyzes the interconversion of CO_2_ and HCO_3_^-^ (Occhipinti and Boron, 2019). In addition, the biochemical reactions of the metabolism of the bacterial cells involve a turnover of protons, which subsequently influences the pH (Ratzke and Gore, 2018). The analysis was performed using the old Wilber-Andersont (WA) method (Wilbur and Anderson, 1948) which is still used (Mustaffa et al., 2017). The principle of the methods is based on tracking the time taken by the enzyme to catalyze the hydration of CO_2_. The hydration activity is monitored by the release of protons (H^+^) during the reaction, which significantly lowers the pH of the solution. The esterase and hydration activities associated to the bacterial cells and those released in the supernatant of the cultures were followed during growth. Preliminary results (not shown) evidenced a maximum of activity associated to cells or in the supernatant, obtained after 72 h incubation, which also corresponded to the exponential growth phase of the bacterial cells. Both activities dropped beyond 80 h incubation, corresponding to the deceleration phase of growth before entering in the stationary phase. The carbonic anhydrase, if any, is then a primary metabolite. Both activities were calculated as specific arbitrary units (AU/10^8^ cfu), to evaluate the potential of each strain.

The isolated strains from the marine sediments and strains of *Virgibacillus*/*Bacillus,* previously isolated from decaying mats of Dohat Faishakh Sabkha in addition to the strains isolated from soils were used in this study. Indeed, we used four *Virgibacillus* strains (DF112, DF251, DF291 and DF2141) reported to have significant mineralizing potentials (Al Disi et al., 2017), while two *B. lichenifomis* (DF141and S33), one *B. cereus* (6.8) and one *Virgibacillus* (Z7D1) were used as non-mineral forming strains. From the collection of isolated strains from the marine environment, mineral-forming strains, *Psychrobacter* sp. (RS5) and *Vibrio alginolyticus* (RS3) were tested for esterase and hydration activities, in addition to *Exiguobacterium arantiacum* (RS1) as one of the non-mineral-forming strains. In literature, none of these bacterial species was previously investigated for esterase activity, making this data novel and open to further studies.

It is clear that the mineral forming strains have significantly higher esterase activities than the non-forming ones. However, the *Vibrio alginolyticus* (RS3) exhibited extremely low extracellular esterase activity. Instead, significant proton generating activities were recorded for the mineral forming strains compared to the non-mineral forming strains. Both *Virgibacillus* strains (DF112) and (DF2141) recorded the highest intracellular hydration specific activities compared to the other studied strains as well as to the extracellular specific hydration activities. These results are consistent with reports confirming the role of carbonic anhydrase hydration activities in biomineralization (e.g. Bose and Satyanarayana, 2017; Bhagat et al., 2017; Görgen et al., 2021). Moreover, the intracellular esterase and intracellular hydration activities have higher values than those of extracellular specific activities, which may indicate a critical role for the microbial cells and also the cells membrane in providing nucleation sites and facilitating the formation of carbonate minerals (Zhu and Dittrich, 2016; Anbu et al., 2016; Ortega-Villamagua et al., 2020).

Rodriguez-Navarro et al*.* (2019) evidenced multiple roles for the carbonic anhydrase enzyme. In addition to carbonic anhydrase role in catalyzing the reversable hydration reaction of CO_2_ to HCO_3_^−1^ that is rate determining for the precipitation and dissolution of calcium carbonate, the carbonic anhydrase enzyme accelerates the precipitation of metastable amorphous calcium carbonates and their consequent conversion into crystalline calcite (Rodriguez-Navarro et al., 2019). In some cases, the carbonic anhydrase activities may be up regulated upon the increase of calcium concentration and thus result in the dissolution of calcite (Zhu et al., 2017). *Virgibacillus* strains mediate the formation of carbonate minerals with high magnesium content. *Virgibacillus* strains (DF112 and DF2141) recorded significant higher hydration activities than the non-forming ones. Although, there is a negative but not significant correlation (p-value > 0.05) between the hydration activity and the amount of incorporated magnesium, however no clear relationship could be established in this study.

Although the bacterial genus *Vibrio* have not been reported to have a potential of biomineralization, however, several of its species are known to cause infections in fish and other marine species. Interestingly, strains of *V. alginolyticus* were reported to cause coral PAWS (*Porites andrewsi* White Syndrome) in 2013 in the South China Sea. When the coral, *Porites andrewsi* is infected by this bacterium, it undergoes coral bleaching and its white calcium carbonate skeleton becomes visible (Zhenyu et al., 2013). To date, little is known regarding the mechanism of infection by *V. alginolyticus*, however, it is suggested that development of further detection methods of this species could possibly help in avoiding the loss of the coral reef. It was hypothesized earlier, that *Vibrio* species are able to adhere to the corals and somehow penetrate its skeletal tissues (Ben-Haim and Rosenberg, 2002). With these findings regarding the ability of *V. alginolyticus* to form calcium carbonates, it is possible that it is a mechanism linked to the infection it causes in coral reef species.

## 5 Conclusion

In this study, the role of carbonic anhydrase enzyme in formation of carbonate minerals was elucidated. A collection of bacterial strains isolated from sea sediments, Sabkhas and soils was used to evaluate the esterase and hydration activities in relation to carbonates formation by the mineral-forming strains, as well as in the non-forming ones. Interestingly, one *Pyschrobacter* sp*.* was isolated, for the first time, in a region known with hot sea water. As expected, this *Psychrobacter* sp. strain RS5 was shown to mediate carbonate-formation. Two strains of *Vibrio alginolyticus* (RS9) and *Vibrio alginolyticus* (RS3) are interesting because they are originated from Qatar coral reef, and can form minerals, significantly. The strains originated from Sabkhas are mostly belonging to *Virgibacillus*, strongly involved in minerals deposition. All the mineral-forming strains exhibited, significantly, high esterase and hydration activities compared to the non-forming ones. This is mostly observed with strains of *Virgibacillus* spp.*, Vibrio alginolyticus* and *Pyschrobacter* sp*.* The non-mineral forming strains recorded very low esterase and hydration activities. Considering both activities (mineral formation and carbonic anhydrase activities), one can conclude that a strong potential exists with bacteria exhibiting high carbonic anhydrase activities to form carbonate minerals.

## Declarations

### Author contribution statement

Rim Abdel Samad: Performed the experiments; Analyzed and interpreted the data; Wrote the paper.

Zulfa Ali Al Disi: Conceived and designed the experiments; Analyzed and interpreted the data; Wrote the paper.

Nabil Zouari: Conceived and designed the experiments; Analyzed and interpreted the data; Contributed reagents, materials, analysis tools or data; Wrote the paper.

Mohammed Abu-dieyeh and Mohammad A. Al-Ghouti: analyzed and interpreted the data.

### Funding statement

This work was supported by the QUST-1-CAS-2020-9 fund, a student grant from Qatar University. This publication was made possible by PDRA Grant No. PDRA5-0425-19007 from the Qatar National Research Fund (a member of Qatar Foundation). The statements made herein are solely the responsibility of the authors. Open Access provided by Qatar University and the Qatar National Library.

### Data availability statement

Data included in article/supplementary material/referenced in article.

### Declaration of interests statement

The authors declare no conflict of interest.

### Additional information

No additional information is available for this paper.
